# Telomere shortening accelerates tumor initiation in the L2-IL1B mouse model of Barrett esophagus and emerges as a possible biomarker

**DOI:** 10.18632/oncotarget.28198

**Published:** 2022-02-14

**Authors:** Vincenz Sahm, Carlo Maurer, Theresa Baumeister, Akanksha Anand, Julia Strangmann, Roland M. Schmid, Timothy C. Wang, Michael Quante

**Affiliations:** ^1^II Medizinische Klinik, Technische Universität München, Munich, Germany; ^2^Department of Medicine, Columbia University Irving Medical Center, New York, NY, USA; ^3^Klinik für Innere Medizin II, Universitätsklinikum Freiburg, Freiburg, Germany

**Keywords:** Barrett's esophagus, telomere shortening, esophageal cancer, risk factor, TERT/TERC

## Abstract

Barrett’s esophagus (BE) is a precursor of the esophageal adenocarcinoma (EAC). BE- development and its progression to cancer is associated with gastroesophageal reflux disease. However, there is currently no molecular risk prediction model that accurately identifies patients at high risk for EAC. Here, we investigated the impact of shortened telomeres in a mouse model for Barrett esophagus (L2-IL1B). The L2-IL1B mouse model is characterized by IL-1β-mediated inflammation, which leads to a Barrett-like metaplasia in the transition zone between the squamous forestomach and glandular cardia/stomach. Telomere shortening was achieved by mTERC knockout. In the second generation (G2) of mTERC knockout L2-IL1B.mTERC^−/−^ G2 mice exhibited telomere dysfunction with significantly shorter telomeres as measured by qFISH compared to L2-IL1B mice, correlating with stronger DNA damage in the form of phosphorylation of H2AX (γH2AX). Macroscopically, tumor area along the squamocolumnar junction (SCJ) was increased in L2-IL1B.mTERC^−/−^ G2 mice, along with increased histopathological dysplasia. *In vitro* studies indicated increased organoid formation capacity in BE tissue from L2-IL1B.mTERC^−/−^ G2 mice. In addition, pilot studies of human BE-, dysplasia- and EAC tissue samples confirmed that BE epithelial cells with or without dysplasia (LGD) had shorter telomeres compared to gastric cardia tissue. Of note, differentiated goblet cells retained longer telomeres than columnar lined BE epithelium. In conclusion, our studies suggest that shortened telomeres are functionally important for tumor development in a mouse model of BE and are associated with proliferating columnar epithelium in human BE. We propose that shortened telomeres should be evaluated further as a possible biomarker of cancer risk in BE patients.

## INTRODUCTION

Esophageal adenocarcinoma (EAC) is on the rise in western countries with increased incidence and high mortality [1, 2]. Barrett’s esophagus (BE) has been identified as a premalignant condition with the ability to progress through stages of low-grade dysplasia (LGD) and high-grade dysplasia (HGD) to EAC [3, 4]. Therefore, increasing focus has been set on the precursor lesions within BE, and on finding new biomarkers for clinical risk prediction [5]. Throughout neoplastic progression, genetic alterations of tumor suppressors p16 and p53, copy number alterations (CNA) and DNA aneuploidy can be observed [6–8], likely facilitated by increased chromosomal instability [9]. Of note, EAC shows a remarkable resemblance to chromosomal instability (CIN) gastric cancer, which is also characterized by chromosomal instability [10]. Furthermore, mutation patterns suggest that a significant share of EACs underwent “genomic catastrophes” in the form of chromothriptic events and breakage-fusion-bridge (BFB)-cylces [11]. Both of which have been linked to CIN caused by telomere dysfunction [11, 12].

Chromosomal stability can be impaired in humans when telomere function is abrogated, which normally occurs with aging after a critical amount of cell divisions [13]. Telomeres are terminal repetitive DNA sequences at the ends of each chromosome that, together with associated proteins, form a protective structure and prevent chromosome degradation, recombination, and fusion. Telomere shortening can lead to the loss of this protective function, which may entail end-to-end chromosomal fusions followed by anaphase bridges, chromosomal breakage, and repetitive BFB-cycles [14, 15]. Dysfunctional telomeres have a complex role on oncogenesis, which has been demonstrated in a mouse model that lacks the telomerase subunit TERC, leading to progressive telomere shortening and dysfunctional telomeres in later generations [16]. While short telomeres alone in these late-generation mTERC-null mice can rescue the phenotype in cancer-prone mouse models [17–19] and therefore act as a tumor suppressor, mild telomere dysfunction has been shown to be associated with tumor initiation [17, 20, 21].

Shortened telomeres is a common sight in epithelial cancers and has also been described in EAC and its precancerous lesions. Specifically, a study by Finley et al. [22] has shown that epithelial cells of all histologically discernable precancerous lesions of EAC (BE, LGD and HGD) feature shortened telomeres compared to healthy gastric tissue. Here, telomere shortening was strongest in BE and decreased with increasing levels of dysplasia, which prompted the idea of dysfunctional telomeres in the early stages of malignant transformation and telomere length stabilization at later stages. Furthermore, evidence of chromosomal instability in the form of abnormal chromosome count has been shown to accompany shortened telomeres. Another study on human BE samples with the very precise STELA-method has shown telomere lengths to be in a range in which telomere fusion is likely and a predictor of worse survival rates in other cancer types [23].

Thus, both CIN and telomere shortening were found to be early events in the neoplastic progression of BE, but whether telomere shortening contributes directly to cancer development has not been examined.

Here we aimed to provide functional evidence for the hypothesis that telomere shortening can directly contribute to tumor initiation, and thus serve as a potential biomarker for BE cancer risk stratification [22, 24]. In order to evaluate the impact of telomere shortening on early stages of metaplasia in BE, we eliminated mTERC (mTERC^−/−^) the catalytic subunit of telomerase [25] in the L2-IL1B mouse model of BE [26].The IL1B-mouse model closely resembles human disease, leading to a Barrett-like metaplasia at the gastroesophageal junction with progression from BE to LGD to HGD and EAC.

Furthermore, we investigated telomere properties in human tissue samples regarding their potential utility as a biomarker. For this we measured telomere lengths using FISH and accounted for the metaplastic mosaic of Barrett’s esophagus [27] by discerning the epithelial fraction into mucus cells and non-mucus cells.

## RESULTS

### Telomere shortening accelerates tumor initiation in the L2-IL1B mouse model

**Figure 1:**
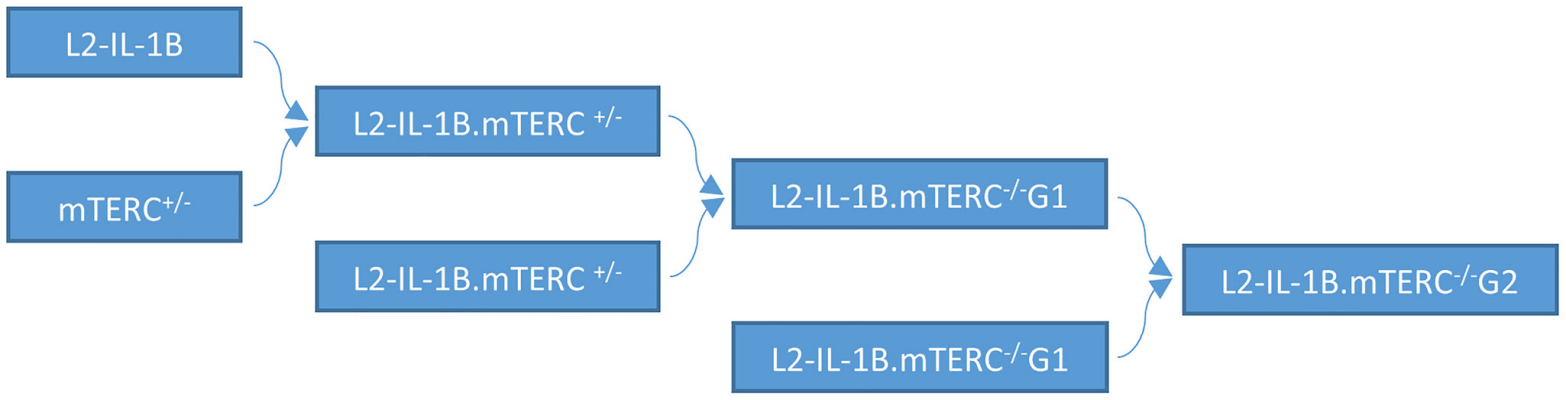
Mouse breeding pattern. IL-1B mice were mated with mTERC^+/−^ mice. Offspring with the genotype L2-IL-1B.mTERC^+/−^ were mated to obtain IL-1β mice with with mTERC- knockout in the first generation: pL2-IL-1B.mTERC^−/−^ G1. Mating of these mice (L2-IL-1B.mTERC^−/−^ G1) yielded L2-IL-1B.mTERC^−/−^ G2 mice.

**Figure 2:**
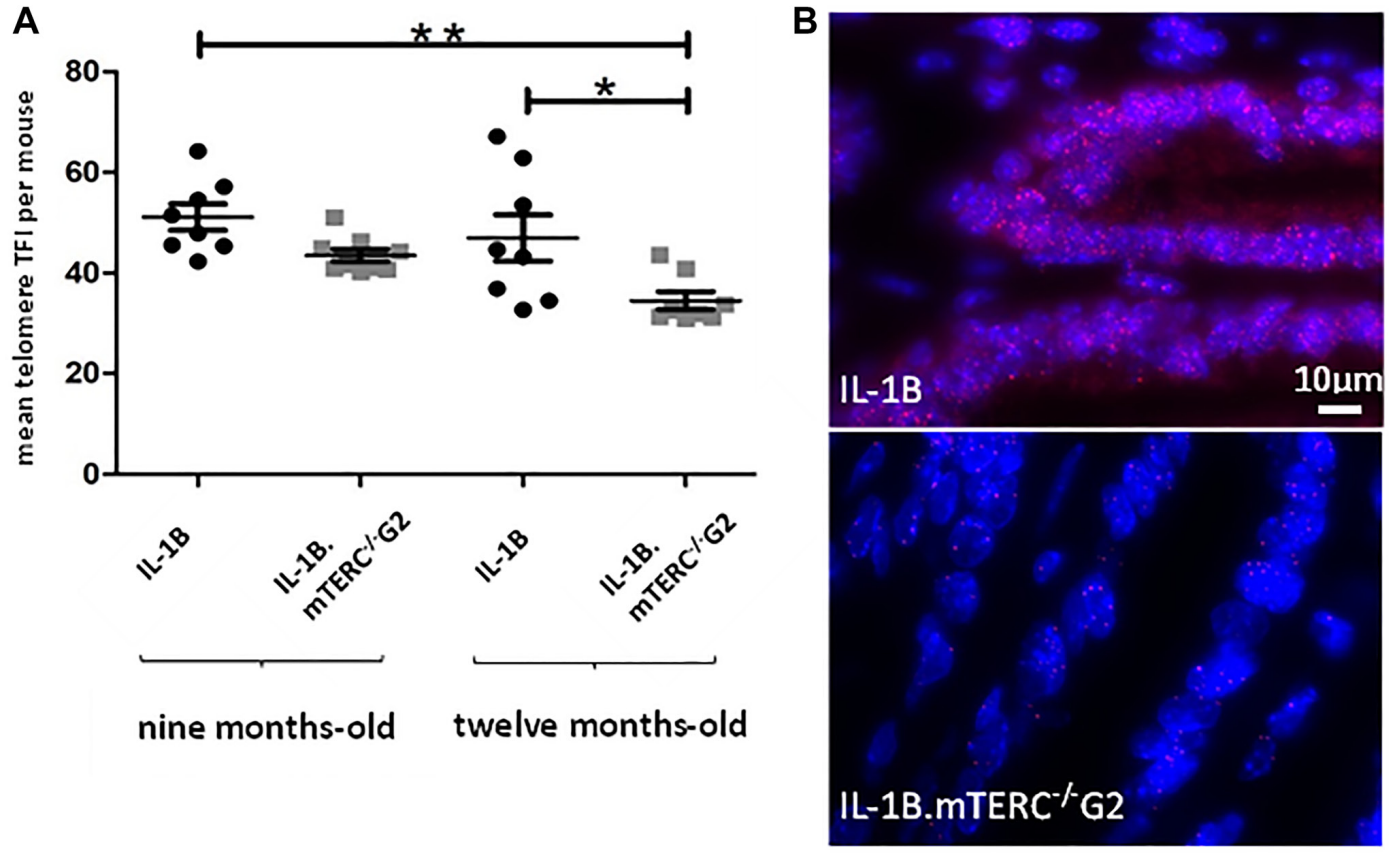
Murine telomere analysis. Figure (**A**) depicts telomere length as mean TFI of SCJ-cells per mouse of both genotypes at both time points. Means differed significantly between 9-month-old L2-IL-1B mice and 12-month-old L2-IL-1B.mTERC^−/−^ G2 mice (*p* < 0.01, one-way-ANOVA, Tukey’s post-hoc test). Furthermore means of 9-month-old L2-IL-1B.mTERC^−/−^ G2 mice and 12-month-old L2-IL-1B.mTERC^−/−^ G2 mice differed significantly (*p* < 0.05, one-way ANOVA, Tukey’s post-hoc test). Graphic (**B**) shows exemplary qFISH-images of both genotypes. Red spots show Cy3-labeled telomeres; DNA is stained blue by DAPI.

The importance and effect of shortened telomeres was investigated in the L2-IL1B mouse model [26] after backcrossing to mTERC^−/−^ mice [28] to generate second generation L2-IL1B.mTERC^−/−^ G2 mice (Figure 1). This genotype was then compared to the original L2-IL1B model. Telomere lengths were measured *in-situ* using telomere-FISH on metaplastic murine SCJ-tissue. We compared telomere lengths of both genotypes at an early stage of disease at nine months of age and at a later time point (12 months). Measurements confirmed- as was expected- shorter telomeres in IL1B.mTERC^−/−^ G2 mice at both time points: at 9 months: 51.2 ± 18.3 SD (L2-IL1B) vs. 43.5 ± 15.1 SD (L2-IL1B.mTERC^−/−^ G2); *p* < 0.01, unpaired *t*-test and at 12-months 43.5 ± 20.9 SD (L2-IL1B) vs. 34.3 ± 14.2 SD (L2-IL1B.mTERC^−/−^ G2, Figure 2A). Figure 2B shows exemplary FISH stainings of both genotypes and illustrates the differences in telomere length.

**Figure 3:**
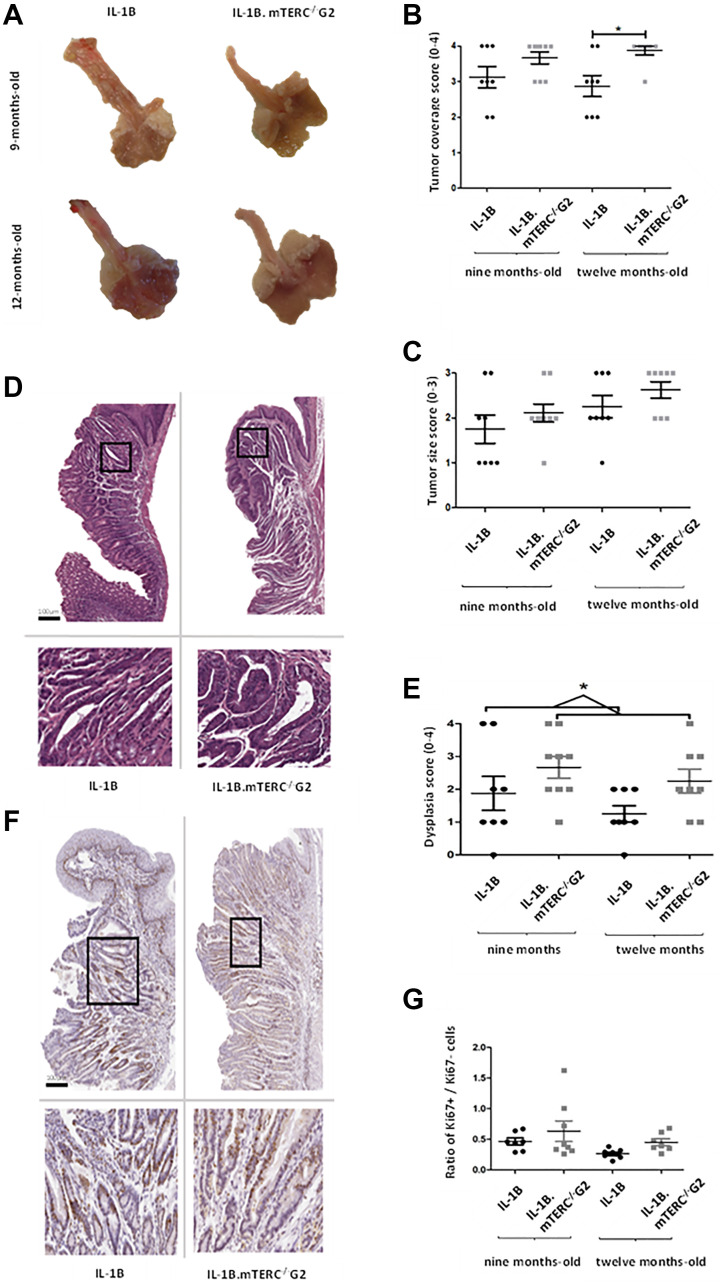
Macroscopic and microscopic comparison of murine tumors. Figure (**A**) shows exemplary images of processed/opened murine stomachs with attached esophagi of both genotypes at both time points. These images were subject to macroscopic analysis with assessment of tumor coverage and tumor size. (**B**) depicts scores for tumor coverage of both genotypes at both time points. Means and standard deviation are indicated by horizontal bars, the asterisk marks a significant difference. The 12-months-old L2-IL-1B.mTERC^−/−^ G2 group exhibits significantly stronger tumor coverage than the 12 months L2-IL-1B group (*p* = 0.02, one-way ANOVA, Tukey’s pos*t*-test). In graphic (**C**) scores for tumor size of both genotypes and time points are displayed, means and standard deviation are indicated by horizontal bars. There is a trend of larger tumors in L2-IL-1B.mTERC^−/−^ G2 mice at both time points, however no significant difference (*p* = 0.11, one-way ANOVA, Tukey’s pos*t*-test). Graphic (**D**) shows exemplary scans of H&E-stained murine SCJ-tissue of both at the 12-months-time-point. Framed areas in the upper scans are enlarged below. Graphic (**E**) shows dysplasia scores of SCJ-regions of both genotypes at both time points. There was a trend of stronger dysplasia in each L2-IL-1B.mTERC^−/−^ G2 group compared to their age matched L2-IL-1B control group. However with the limited number of observations a significant difference was only seen when scores at both time points were pooled (*p* = 0.02, unpaired *t*-test). Graphic (**F**) shows exemplary scans of SCJ-tissue of both genotypes at the 12-months time point stained for Ki67. The upper images contain the whole SCJ with stratified esophageal epithelium at the top and columnar cardiac/gastric epithelium below. Framed areas, which contain cardiac glands are magnified in the images below. Dark brown stained nuclei show Ki67 positive cells. In Graph (**G**) ratios of Ki67- positive epithelial cells to unstained epithelial cells (Ki67 negative) for groups subdivided by genotype and time point are plotted. Means and standard deviation are indicated by horizontal bars. There were no significant differences between the 4 groups (*p* < 0.08, one-way ANOVA, Tukey’s post-hoc test).

Macroscopic tumor formation was assessed in view of overall tumor area along the SCJ and size of individual tumors at an early stage (9 months) of tumorigenesis (L2-IL1B: *n* = 8; L2-IL1B.mTERC^−/−^ G2: *n* = 9) and at a later stage (12 months; L2-IL1B: *n* = 8; L2-IL1B.mTERC^−/−^ G2: *n* = 8) and showed significantly stronger tumor coverage in 12-months-old L2-IL1B.mTERC^−/−^ G2 mice compared to 12 month old L2-IL1B mice (Figure 3A and 3B). With respect to tumor size, tumors in L2-IL1B mice (9 months 1.75 ± 0.89 SD, 12 months 2.25 ± 0,71 SD) trended smaller than those in L2-IL1B.mTERC^−/−^ G2 mice (9 months 2.11 ± 0.60 SD, 12 months 2.63 ± 0.52 SD), although there was no significant difference between the groups (*p* = 0.11, one-way ANOVA, Tukey’s post-hoc test, Figure 3C).

Nevertheless, histological analysis showed an overall increase in dysplasia scores in L2-IL1B.mTERC^−/−^ G2 mice compared to L2-IL1B mice (L2-IL1B 9-months-old 1.88 ± 1.46; L2-IL1B 12-months-old 1.25 ± 0.71; L2-IL1B.mTERC^−/−^ G2 9-months-old 2.67 ± 1.00 L2-IL1B.mTERC^−/−^ G2 12-months-old 2.25 ± 1.04, Figure 3E). Exemplary histological images are presented in (Figure 3D). This trend became significant when time points were pooled to get a comparison between the two genotypes with a larger number of observations: L2-IL1B mice had a mean dysplasia score of 1.56 ± 1.15SD whereas the L2-IL1B.mTERC^−/−^ G2 group had a mean dysplasia score of 2.47 ± 1.01SD (*p* = 0.02, unpaired *t*-test, Figure 3E). In the L2-IL-1B mouse model, the chosen time points at 9 and 12 months of represent early stages of the disease. L2-IL-1B mice without further acceleration (such as HFD [29], bile acid [26], Notch [30], IL-8 [29], NfkB [31]) develop strong dysplasia consistently only at the age of 15 months.

While Ki67 staining revealed strong proliferation in the SCJ area in both genotypes, there was a not significant trend towards higher proliferation rates in L2-IL1B.mTERC^−/−^ G2 mice compared to the L2-IL1B mice both at 9 months (0.61 ± 0.45 SD vs. 0.47 ± 0.14 SD) and 12 months of age (0.45 ± 0.14 vs. 0.27 ± 0.08, *p* < 0.08, one-way ANOVA, Tukey’s post-hoc test, Figure 3F and 3G).

In summary macroscopic and histopathologic tumor scores were increased in L2-IL1B.mTERC^−/−^ G2 mice, with no significant change in individual tumor size, suggesting that genetic shortening of telomeres may be important for tumor initiation early during carcinogenesis but may not promote tumor growth.

### Dysfunctional telomeres induce DNA damage in L2-IL1B.mTERC^−/−^ G2 mice

**Figure 4:**
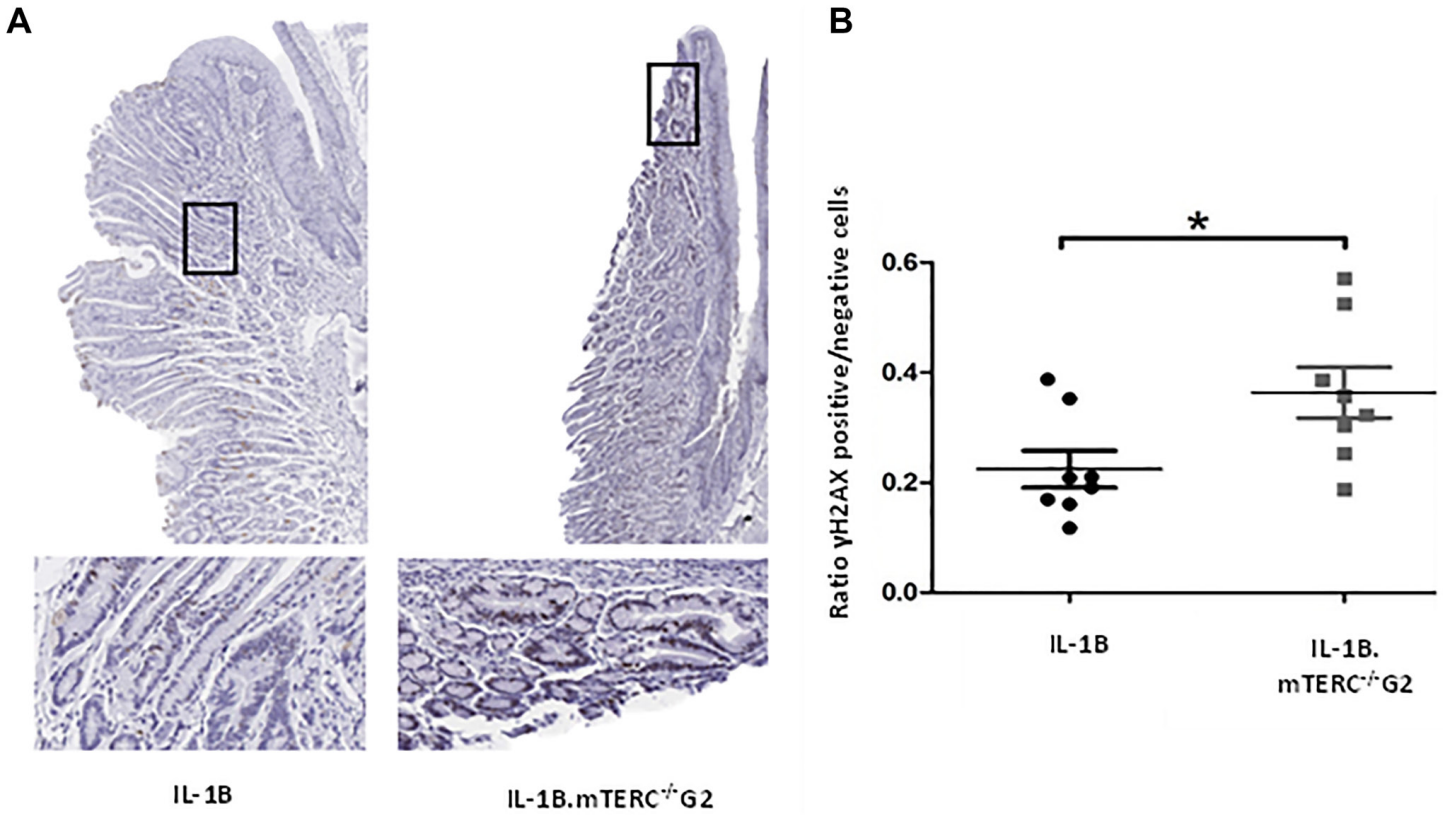
DNA-damage analysis. Graphic (**A**) shows representative scans of SCJ-tissues of 12-month-old L2-IL-1B and L2-IL-1B.mTERC^−/−^ G2 mice stained for yH2AX with framed regions magnified in the images below. Dark brown/black (DAB) stained nuclei indicate antibody-binding at γH2AX loci. Graph (**B**) displays ratios of γH2AX positive to γH2AX negative cells at the SCJ regions of 12 months-old mice of both genotypes. Means and standard deviation are indicated by horizontal bars. The asterisk marks a significant difference. 12 month old L2-IL-1B.mTERC^−/−^ G2 show a significant higher rate of γH2AX positive cells, compared to 12-month-old L2-IL-1B mice (*p* < 0.03, unpaired *t*-test).

Dysfunctional telomeres, either through uncapping in the absence of TRF2 or when reaching a critical length, can lead to phosphorylation of H2AX, which produces γH2AX, a damage response factor and an indicator of DNA double strand breaks (DSB) [32, 33]. In order to evaluate whether the induced shortening of telomeres in BE had a measurable impact on the amount of DNA-damage and -damage response, SCJ-tissues from 12-month-old L2-IL1B.mTERC^−/−^ G2 mice and 12-month-old L2-IL1B mice were assayed for γH2AX-loci (Figure 4). Indeed, L2-IL1B.mTERC^−/−^ G2 mice had a significantly higher mean ratio of γH2AX-positive cells to γH2AX-negative cells in the SCJ-area (0.36 ± 0.13 SD, (*n* = 8)), compared to L2-IL1B mice (0.22 ± 0.09 SD (*n* = 8), *p* < 0.03, unpaired *t*-test, Figure 4). These data point to increased DNA damage as a result of shortened telomeres, which may contribute to early tumorigenesis.

### 3D *in vitro* analysis shows greater organoid formation capacity in L2-IL-1B.mTERC^−/−^ G2 mice

**Figure 5:**
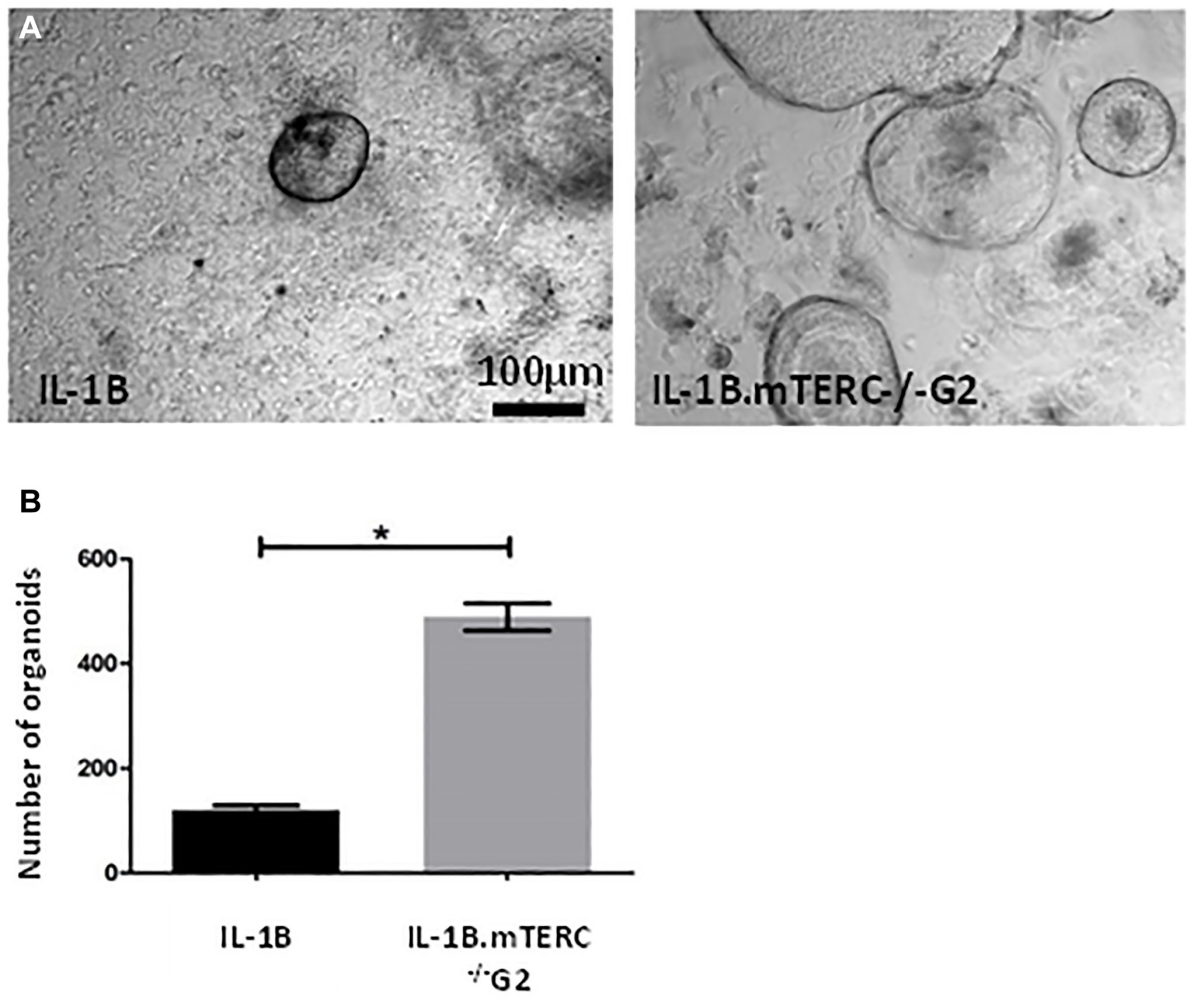
*In-vitro* analysis. (**A**) shows exemplary light microscopic photo of organoids cultivated from SCJ cell isolations. Gross visible comparison yielded no differences in organoid morphology (size, organoid wall thickness). Graph (**B**) depicts the number of organoids 48 h after the 3rd passage for both genotypes after correction for organoid number at the beginning of the 3rd passage. Means and standard error of the means are indicated. The L2-IL-1B.mTERC^−/−^ G2 group presented with a significantly higher number of organoids (*p* < 0.01, unpaired *t*-test).

Additionally, 3D-organoid BE culture was performed to measure *in vitro* growth of dysplastic SCJ cells, which may reflect the impact of genomic instability on progenitor cells within the BE tissue [34]. With respect to the appearance and macroscopic phenotype for BE organoids from the two groups of mice, there were no obvious differences. In the 3rd passage, after normalizing for the number of organoids initially plated, the mean organoid count after 48 h was significantly increased in L2-IL1B.mTERC^−/−^ G2 mice (*n* = 3) compared to L2-IL1B mice (*n* = 3, *p* < 0.01 unpaired *t*-test, Figure 5). This suggests that telomere dysfunction could be seen as an early driver of increased epithelial progenitor cell survival and expansion in the L2-*IL-1B mouse model*.

### Epithelial telomere length is shortened in human BE- and LGD samples

**Figure 6:**
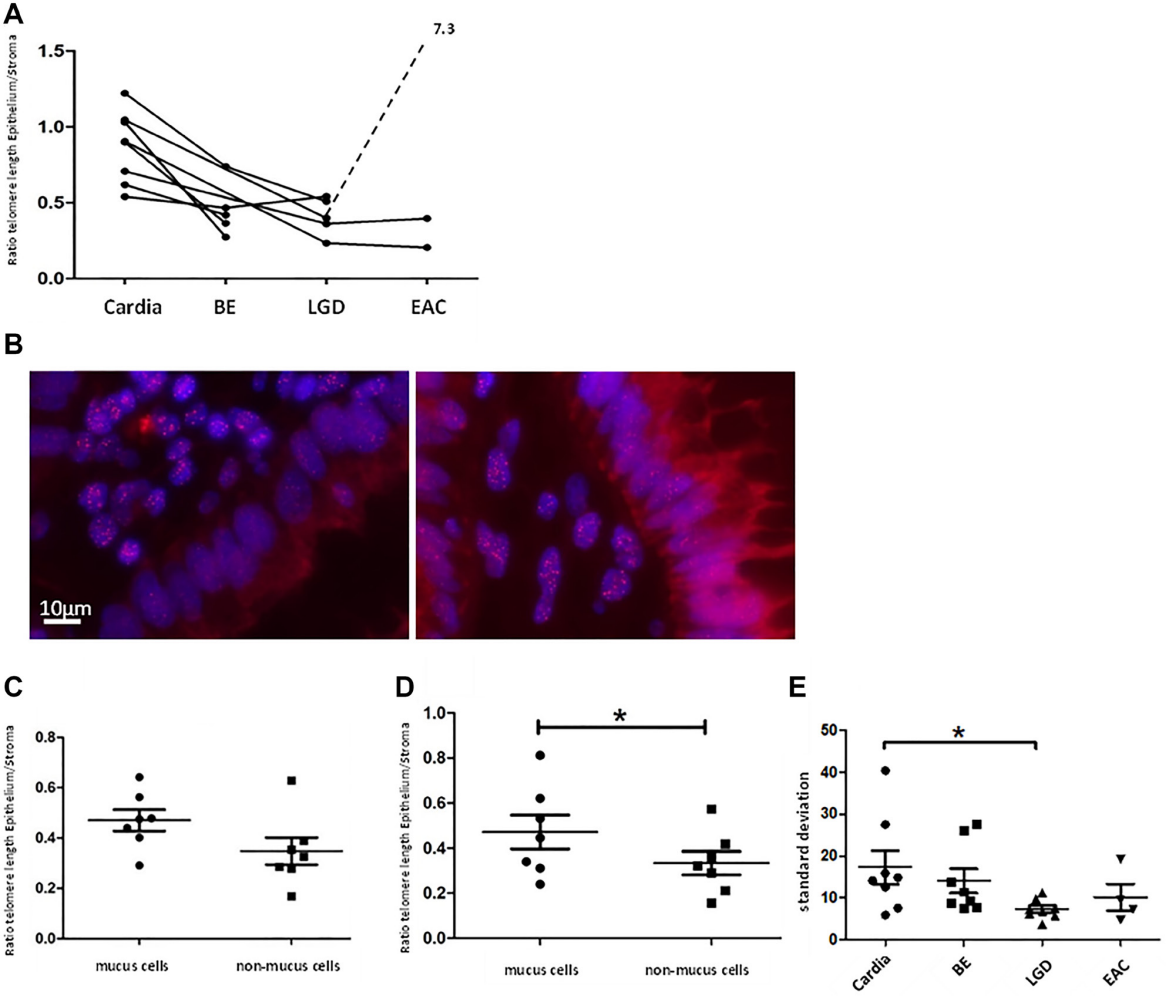
Human telomere analysis. Graph (**A**) shows telomere length according to tissue type, beginning with normal cardia tissue as a reference on the left with advancing stages of BE up to EAC to the right. Each point represents the calculated telomere length ratio of epithelial cells to stromal cells of a histologically defined sample. Connection of points indicates that samples originate from the same patient. We measured significant longer telomeres in cardia tissue compared to BE (*p* < 0.005) and LGD (*p* < 0.001). (**B**) displays two telomere-FISH images of the same human LGD sample. In the bottom right part of the left picture a crypt with cells belonging to the “non-mucus”-group/ columnar lined epithelium is visible, marked by cells in straight array but lack of vacuoles. In the right picture crypt cells possess vacuoles and were therefore grouped as “mucus”/ goblet cells. Figure (**C**) and (**D**) show telomere length as a ratio of epithelial- to stromal- cell telomere length for both types of epithelial cells (mucus and non-mucus) in human BE- and LGD samples. Means and standard deviation are indicated, Asterisk implies significance. In BE samples there is a trend of shorter telomeres in non-mucus cell compared to mucus cells. In LGD-samples this trend becomes more evident (*p* = 0.05, paired *t*-test). (**E**) depicts standard deviation of epithelial cells’ mean telomere lengths calculated per individual biological specimen and tissue type. LGD epithelial cells presented with significantly lower standard deviation compared to cardiac epithelial cells (*p* < 0.03 unpaired *t*-test).

Telomere length measurements of human Cardia-, BE-, LGD-, and EAC samples in our cohort confirmed earlier findings [22] that BE- and LGD- epithelial cells possess significantly shorter telomeres compared to telomeres in cells from healthy gastric cardia tissue (Figure 6A). Of note, we analyzed different areas of BE and LGD in comparison to the gastric cardia, thought to represent the likely origin of BE [26, 35]. Mean telomere length ratio between metaplastic epithelial tissue and stroma was 0.45 ± 0.18 SD for BE (*n* = 5) and 0.41 ± 0.12 SD for LGD (*n* = 5), which was significantly lower (*p* < 0.005 and *p* < 0.001) compared to cardia mean telomere length ratio (0.87 ± 0.23 SD, *n* = 8, Figure 6A). Assuming that BE and esophageal dysplasia arise from the gastric cardia, these data suggest that with accumulating cell divisions without telomerase activation, telomeres in BE metaplasia are shortened over time.

### Mucus producing cells retain longer telomeres in human BE- and LGD samples

Since BE exhibits a metaplastic mosaic [27], with areas of gastric and intestinal metaplasia, we wanted to investigate whether different epithelial cell types present with distinct telomere lengths. Intestinal-like metaplasia appears to be more differentiated, with lower levels of Notch signaling and less proliferation [30, 36]. We therefore divided epithelial cells of BE- and LGD samples whenever possible into two groups: intestinal-like goblet cells, marked by a vacuole like autofluorescence pattern, and columnar lined cells (Figure 6). We analyzed 7 human BE- and 7 LGD samples of which mucus- and non-mucus parts were clearly discernible. In BE samples mucus cells retained longer telomeres compared to non-mucus cells (0.47 ± 0.11 SD vs. 0.34 ± 14 SD) which however was not significant (*p* = 0.08, paired *t*-test, Figure 6C). In LGD samples, mucus cells retained significantly longer telomeres (0.47 ± 0.20 SD vs. 0.33 ± 0.14, *p* = 0.05, paired *t*-test, Figure 6B and 6D). These data, in combination with previous results [30, 36], may suggest that goblet cells, which are terminally differentiated and do not proliferate, are likely not the site of initial telomere shortening that contributes to malignant transformation.

### Cell-to-cell telomere length variation is reduced in human LGD tissues

Another established measure of telomere length in tissues is telomere length variation (TLV), which has been evaluated in other cancer types [24, 37] and proven to be a prognostic marker [24]. In our study we surveyed telomere length variation by comparing standard deviation of cellular telomere length per biological specimen. Indeed, epithelial cardia cells presented with the highest cell-to-cell telomere length variability with a mean standard deviation of 17.39 ± 11.35 SD, *n* = 8 (Figure 6E). All other tissue types presented with less epithelial TLV, with LGD tissue samples showing the lowest TLV, with a mean standard deviation of 7.306 ± 2.35 SD (*n* = 8) which was significantly less compared to cardiac samples (*p* < 0.03, unpaired *t*-test, Figure 6E). This supports the concept that BE- and LGD tissues can lose cell heterogeneity and cells are uniformly losing telomere length, leaving patches of clonal expansion.

## DISCUSSION

Here, we demonstrate that telomere dysfunction aggravates the histological phenotype, extends the tumor area in the inflammation-based L2-IL1B mouse model for BE and acts as a driver for early dysplasia development. Telomere dysfunction in the original mTERC^−/−^ mouse model was seen from generation two onwards, accompanied by signs of chromosomal instability [25]. Accordingly, we assumed that chromosomal instability caused by telomere shortening plays an important role in the L2-IL1B mouse model. In line with this assumption, we demonstrated a significant decrease in telomere length in L2-IL1B.mTERC^−/−^ G2 mice as well as increased phosphorylation of H2AX (γH2AX) in L2-IL1B.mTERC^−/−^ G2 mice compared to their L2-IL1B counterparts, a sign for DNA- double strand breaks which was previously described in mTERC^−/−^ mice [38]. Telomere shortening was shown to lead to p53 mediated senescence [39], which may have limited to some extent the degree of tumor growth in our model. In accordance with this notion, individual tumor sizes in the two mouse lines were comparable, consistent with a p53-mediated break on tumor growth, which is still functional in the *L2-IL1B.mTERC*^−/−^ *G2 model*. Investigations of other cancer-prone mouse models with dysfunctional telomeres have consistently noted impaired tumor development [18, 19, 40, 41] with increased levels of senescence and/or apoptosis. When p53, the main mediator for telomere induced senescence, was abrogated additionally to telomere dysfunction, tumor growth was enhanced [42, 43]. However, it has been shown that telomere dysfunction with intact p53 can be related to increased tumor initiation in some mouse models [17, 21].

While *in-situ* Ki67 assays found no significant differences in proliferation between the two genotypes, *in-vitro* organoid culture showed a marked increase in organoid formation capacity of L2-IL1B.mTERC^−/−^ G2 SCJ-isolates, compared to the L2-IL1B controls. The observed boost in organoid proliferation was likely caused by a process of cell selection in the wake of telomere dysfunction. Accumulating evidence indicates that age dependent (stem-)cell selection can increase cancer risk, with telomere shortening as a possible driver [44, 45]. Furthermore, telomere dysfunction induced clonal dominance of chromosomal unstable cells, albeit in the background of p53 deficiency [46]. Findings in the framework of the human caner genome project have clearly outlined the importance of CIN in the development of EAC [10] and mutational signatures typical of telomere based chromosomal instability [11].

With our telomere length measurements on human samples, we were able to replicate prior findings [47] of telomere shortening in the epithelial compartment of BE and LGD samples. To assess telomere length, we used the ratio of epithelial to stromal telomere lengths. We based this procedure on prior findings that telomere lengths of the stromal compartment are stable across all precancerous lesions of the EAC [47]. The telomere length ratio yields more robust results since it can correct for differences in staining- and general specimen-quality. However, the drawback of this approach is the lost potential to evaluate telomere changes in the stromal compartment. In addition, we compared telomere length in highly differentiated cell types such as goblet cells, compared to less differentiated, non-mucus cells. This analysis demonstrates that in both BE and LGD samples, differentiated goblet cells retained longer telomeres, in contrast to proliferating epithelial cells. This is a remarkable finding since this suggests that mucus-producing cells exhibit a shorter replicative history and are less likely to be the progenitor cells of malignant progression. This would underpin cumulative research that see gastric cardiac cells to give rise to BE and EAC and argue against the transdifferentiation hypothesis in the carcinogenesis of EAC. Although presence of BE with intestinal mataplasia is linked to higher rates of EAC-development, in many cases EAC is diagnosed without the presence of BE. Interpreting our measurements, we see columnar lined epithelium at higher risk of telomere based mutations.

Furthermore, we analysed cell-to-cell telomere length variability in the epithelial cell fraction of our human samples. Telomere length variability has been examined in other cancer types in various approaches [24, 37, 48] and has been proposed as a possible marker for disease progression [24, 48, 49]. For this we calculated standard deviation of cellular telomere length and found it to be decreased in foremost in LGD samples compared to cardia tissue, suggesting monoclonal expansion with uniform telomere lengths in these metaplastic and dysplastic tissue types. Hence, both, differences in overall telomere length between mucus-producing cells (i.e. goblet cells) and non-mucus cells as well as decreased telomere length variability in the epithelial compartment of BE and LGD point to a distinct picture of telomere length dynamics, in contrast to identical telomerase activation in all cell types along the whole metaplastic area in BE [50].

With our patient selection which only included cancer patients we intended to evaluate adjacent to cancer tissue in order to compare it within the same patient, assuming that the development of BE and dysplasia types started at the cardia and would therefore provide a spacial evaluation of a long-term process and therefore giving us the opportunity to evaluate time as well. A drawback of this study design is the fact that we cannot compare progressors and non-progressors with each other, which would be desirable in the evaluation of biomarkers.

In summary, we here demonstrated a functional role of telomere shortening, a well observed property of BE, in promoting early onset esophageal tumor initiation in the L2-IL1B mouse model. Moreover, besides the importance during early carcinogenesis in the mouse model, shortening of telomeres was specifically decreased in dysplastic columnar-type tissue rather than in differentiated goblet cells in human BE- and LGD tissue samples. This underlines our hypothesis that goblet cell differentiation might be a protective feature [30, 36]. Upon verification of such a distinct functional role of telomere length in cell homeostasis, to distinguish proliferating and differentiated cell types our findings may yield a new approach to assessing telomere lengths as a biomarker for malignant progression. Due to the small number of observations in our human group we could find a nonsignificant trend of shorter telomeres in LGD samples compared to BE samples, which demands further larger studies. However our findings of measurably shorter telomeres in the mucus section compared to the non-mucus section in BE and LGD as well as lower epithelial cell-to-cell variability in BE an LGD open new avenues in assessing telomere length in these premalignant tissues. For any given BE tissue we could, for example, measure the telomere ratio between mucus-cells and non-mucus cells and infer a telomere-status which indirectly contains information about the status of intestinal metaplasia. It can be envisioned that the telomere length profile of pre-malignant BE tissue can – together with other biomarkers – estimate the rate of malignant progression. Low cell-to-cell variability in the epithelial compartment may be a surrogate marker for localised clonal expansion. Scientists form another research group who the more precise STELA-method on BE samples argued for local clonal expansions when telomere lengths were within a small range [23]. Maley et al. previously combined genome instability with a measure of clonal expansion and found that both factors together predict progression to EAC better than either factor alone [45]. It is plausible that with our measurements we could emulate this with shortened telomeres being at higher risk of genome instability and lowered cell-to-cell variability marking clonal expansion. However, larger studies are needed to test these hypotheses.

## MATERIALS AND METHODS

### Human biopsies

A total of 25 biopsies were examined from eight patients with EAC (all male, mean age 70.6 ± 8.8 SD years) who underwent full-length esophagectomy. Tissue samples were procured along the entire length of the esophagus and processed to FFPE-blocks and cut into 5 μm slices for H&E staining. These specimens were then scored by an experienced pathologist and categorised according to histology as cardiac-, BE-, LGD- or EAC tissue. We obtained a minimum of three samples per patient: one cardia biopsy as the reference for uncompromised glandular tissue, and two additional samples (three in one case) classified as either BE, LGD or EAC. These samples were subjected to qFISH using specific telomere-binding probes and subsequent telomere analysis.

### Mouse models

All experimental animal work performed in Germany was carried out with the approval of the Regierung Oberbayern according to the animal experimental permits (Tierversuchsanträge) 55.2.1.54-2532-125-12 and 55.2-1-54-2532-24-2016. L2-IL1B mice express human IL-1β under the control of the EBV-L2 promoter leading to BE and EAC through continuous inflammation in the esophagus [26]. L2-IL1B mice were backcrossed more than 8 generations with C57BL/6J mice (wildtype = wt). Telomere shortening was then introduced to this model by crossing to telomerase-deficient mTERC^−/−^ mice [28], also in a C57BL/6 background, to generate L2-IL-1B.mTERC^−/−^ mice. Intercrossing of the latter yielded L2-IL-1B.mTERC^−/−^ mice in the second generation (G2). After weaning and genotyping, L2-IL-1B mice with (L2-IL-1B.mTERC^−/−^ G2) and without telomere deficiency (L2-IL-1B)) were assigned to experimental cohorts. The two genotypes were compared at nine and twelve months of age, representing early and later stages of disease.

### Tissue preparation and disease evaluation

At the time of necropsy, the stomachs with attached lower esophagi were removed. For macroscopic scoring, the stomachs were cut open along the greater curvature, the attached esophagi opened longitudinally and then flattened for photographic documentation. Macroscopic scoring of the squamocolumnar junction (SCJ) and the esophagus was performed following previously reported scoring system for dysplasia assessment in mice [26, 29]. This scoring system encompasses tumor area, individual tumor size and total tumor size. Mouse tissues were fixed in formalin and paraffin-embedded, then cut into 2 μm thick sections and stained with standard H&E (Haematoxylin and Eosin), PAS (periodic acid-Schiff) and Alcian blue. Histopathology was evaluated, blinded to genotype and age, by an experienced mouse pathologist based on a previously described scoring system. Criteria were inflammation, metaplasia and dysplasia [26]:

- Inflammation was scored by the percentage of different immune cells (mostly neutrophilic myeloid cells) in a defined tissue area of the SCJ in a high-power field evaluation.
- Metaplasia was evaluated by the abundance of mucus producing cells per gland and the abundance of glands with mucus producing cells in the BE area at the SCJ.
- Dysplasia was assessed by the amount of cellular atypia and the presence of low- or high-grade dysplasia in single or multiple glands as defined by an experienced mouse pathologist.

### Immunohistochemistry (IHC)

Standard immunohistochemical staining procedures were used, starting with citrate buffer antigen retrieval (1.00244.1000, Merck) followed by staining with the following primary antibodies: Ki67 (Abcam, ab15580, 1:2000) or *γH2AX* (Abcam, ab 26350). After incubation with primary antibodies for 1h (Ki67) or overnight (*γH2AX*) at room temperature and washing steps, sections were incubated with secondary antibodies for 30 min. Antibody binding was visualized with DAB. Three to four regions of esophagus/ stomach tissue per mouse and three to 12 mice per treatment were assessed. For quantification, the percentage of positive cells (i.e. with strong antigen expression) in BE regions was calculated using the cell-counter tool of ImageJ [51]. The BE region was defined as the region between squamous epithelium and oxyntic mucosa of the stomach. Statistical analysis was performed with GraphPad Prism version 5.0 for Windows, GraphPad Software, San Diego, California USA.

### Organoid- *in-vitro* analysis

*In vitro* analysis was executed using organoid culture according to a protocol that we have established for the L2-IL1B mouse model [34]. Tumorous GEJs of freshly sacrificed mice were cut off, disintegrated and filtered to gather cell isolates. Cells were then cultured using a 3D-Medium (Matrigel^®^) and growth factors which leads to 3D cell-organoids. Organoids were then analyzed after their 3rd passage to eliminate confounding effects that occur directly after cell isolation. Comparison of organoid forming capacity was investigated as a measure of proliferation. We determined an organoid starting count 24 h after the 3rd passage and then normalized our data by calculation of an individual factor that yields 100 when multiplied with the organoid starting count. Organoid numbers of both genotypes were compared 96 h after the 3rd passage.

### Telomere FISH staining

Slides were deparaffinized and rehydrated using xylene and ethanol of different concentrations. Antigen retrieval was achieved by a combination of heat-induced and proteolytic-induced epitope retrieval with several washing steps in between and after. For telomere binding we used a telomere-specific peptide nucleic acid (PNA) probe with the N-terminal covalently linked to Cy3. Samples were counterstained with DAPI.

### FISH- image acquisition

Images were taken immediately after staining with Zeiss fluorescence microscope (ZEISS Axioscope 5) at 100x magnification. Optimal exposure time for DAPI- and Cy3-images was determined by the Axio-Vision software belonging to the microscope and was held constant for all human samples and all murine samples respectively. Telomere-specific FISH signals are linearly proportional to telomere length and therefore, telomere length can be quantified via digital image analysis [52] Telomere length was assessed, on a per cell basis, as the ratio of the total intensity of telomeric signals in each cell to the total intensity of the DAPI stained nuclear DNA signal in the same cell. Telomere length assessment—The digitized fluorescent telomere FISH signals were quantified using the open source, JAVA-based image analysis software ImageJ and a custom designed plugin called “Telometer” [53]. This program uses matching telomeric and nuclear DNA grayscale TIFF image file. The images are normalized by simple background subtraction, subsequently run through a sharpening filter, followed by enhancement using a rolling ball algorithm for contouring of telomeric spots. A binarized mask of the telomere signals is then created and applied to the original unfiltered Cy3 telomere fluorescence image for data extraction. For each cell group, a region of interest was manually defined on the DAPI image by use of the freeform drawing tool in ImageJ. Guidance for cell type selection was provided by comparison to a separate two-color merged image showing the combined DAPI and the telomere stain. Cells were classified as epithelial when aligned in a typical crypt like structure, whereas stroma cells were identified by lack of formation. Telomeric signals identified by the binary segment mask, which were contained within the area inscribed by each circled nuclear DNA (DAPI) signal area, were then measured, and the data for each telomeric spot was tabulated.

### Telomere length analysis

Telomere length was evaluated per cell with a single value. Telomeric spots were assessed by their Cy3- fluorescence intensities multiplied by their corresponding areas which yields a quantitative value for telomere length in the form of telomere fluorescence intensity (TFI). Then telomeric signals were normalized by factoring in the intensity of the corresponding DAPI signal. In human samples, cells could be differentiated by their orientation to one another and a distinct autofluorescence pattern. Hence, cells were assigned to the stromal- or the epithelial category, the latter consisting of two sub-groups: mucus producing cells and non-mucus cells. This allows the calculation of a telomere length ratio of epithelial to stromal cells. Hereby each epithelial cell’s telomere length value was divided by the mean telomere length value of all stromal cells in the same image. Furthermore, telomere lengths of mucus producing cells and non-mucus cells could be compared. In murine samples stromal and epithelial cells as well as mucus and non-mucus cells could not be discerned from one another with sureness which only permitted a telomere length comparison between individual biological specimen. For this telomere lengths of order to obtain telomere length distributions on a “mouse-level” the mean TFI of all measured SCJ-cells of one mouse was calculated.

## Abbreviations

3D: three-dimensional
(γ)H2AX: (phosphorylated) H2A histone family member X
BE: Barrett’s esophagus
BFB-cycles: breakage-fusion-bridge cycles
Cy3: tetramethylrhodamine
CAN: copy number alterations
CIN: chromosomal instability
DAB: 3,3′-diaminobenzidine
DAPI: 4′,6-diamidino-2-phenylindole
DNA: deoxyribonucleic acid
EAC: esophageal adenocarcinoma
DSB: double strand break
FFPE: formalin-fixed paraffin-embedded
qFISH: quantitative fluorescence *in-situ* hybridization
G2: second generation
H&E: haematoxylin and eosin
HGD: high grade dysplasia
IL-1β: Interleukin 1 beta
PNA: peptide nucleic acid
SCJ: squamocolumnar junction
(m)TERC: (murine) Telomerase RNA component
TFI: telomere fluorescence intensity
TIFF: Tag Image File Format
LGD: low grade dysplasia
